# Mechanisms of Nrf2 Protection in Astrocytes as Identified by Quantitative Proteomics and siRNA Screening

**DOI:** 10.1371/journal.pone.0070163

**Published:** 2013-07-29

**Authors:** James A. Dowell, Jeffrey A. Johnson

**Affiliations:** 1 Division of Pharmaceutical Science, University of Wisconsin-Madison, Madison, Wisconsin, United States of America; 2 Waisman Center, University of Wisconsin-Madison, Madison, Wisconsin, United States of America; 3 Molecular and Environmental Toxicology Center, University of Wisconsin-Madison, Madison, Wisconsin, United States of America; 4 Center of Neuroscience, University of Wisconsin-Madison, Madison, Wisconsin, United States of America; Laurentian University, Canada

## Abstract

The Nrf2 (NF-E2 related factor 2)-ARE (antioxidant response element) pathway controls a powerful array of endogenous cellular antioxidant systems and is an important pathway in the detoxification of reactive oxygen species (ROS) in the brain. Using a combination of quantitative proteomics and siRNA screening, we have identified novel protective mechanisms of the Nrf2-ARE pathway against oxidative stress in astrocytes. Studies from our lab and others have shown Nrf2 overexpression protects astrocytes from oxidative stress. However, the exact mechanisms by which Nrf2 elicits these effects are unknown. In this study, we show that induction of Nrf2 reduces levels of reactive oxygen species (ROS) produced by various oxidative stressors and results in robust cytoprotection. To identify the enzymes responsible for these effects, we used stable isotope labeling by amino acids in cell culture (SILAC) and quantitative shotgun proteomics to identify 72 Nrf2-regulated proteins in astrocytes. We hypothesized a subset of these proteins might play a critical role in Nrf2 protection. In order to identify these critical proteins, we used bioinformatics to narrow our target list of proteins and then systematically screened each candidate with siRNA to assess the role of each in Nrf2 protection. We screened each target against H_2_O_2_, *tert*-butyl hydroperoxide, and 4-hydroxynonenal and subsequently identified three enzymes–catalase, prostaglandin reductase-1, and peroxiredoxin-6–that are critical for Nrf2-mediated protection in astrocytes.

## Introduction

Oxidative stress has been implicated as a causative agent in a wide spectrum of neurodegenerative diseases, including Alzheimer’s disease, Parkinson’s disease, and amyotrophic lateral sclerosis [1], [2], [3]. In the brain, the activation of the Nrf2-ARE pathway is protective against various stressors, including glutathione depletion, peroxides, excitotoxins, mitochondrial toxins, and intracellular calcium overload [4], [5], [6], [7], [8], [9], [10]. The pathway is composed of an enhancer element, the antioxidant response element (ARE), and its transcription factor, NF-E2-related factor-2 (Nrf2) [4], [11], [12], [13]. Nrf2 is regulated by its binding partner Kelch ECH associating protein 1 (Keap1) [14], [15], [16] and under normal conditions is sequestered by Keap1 in the cytoplasm; however, under conditions of oxidative stress, Nrf2 is released from Keap1 and translocates to the nucleus where it binds the ARE and drives gene expression [17]. Genes under the putatiave control of the Nrf2-ARE pathway are direct antioxidants, including glutathione [18], [19]; enzymes that inactivate ROS, including superoxide dismutase and catalase [20], [21]; reductive co-factors, including NADPH, [22]; and enzymes involved in protein turnover and homeostasis [23].

*In vitro* studies have indicated that the Nrf2 pathway is relatively unresponsive in neurons but highly inducible in astrocytes. Furthermore, astrocytic-specific Nrf2 activation confers protection against ROS to co-cultured neurons [6], [7], [9]. Astrocyte-specific overexpression *in vivo* has been shown to mitigate disease pathogenesis in animal models of Huntington’s disease, amyotrophic lateral sclerosis, Parkinson’s disease, and Alexander’s disease [9], [24], [25], [26].

In primary astrocyte cultures, gene expression profiling has revealed a number of Nrf2-regulated cellular defense pathways including those involved in the production and utilization of glutathione [4], [6], [7]. Additional studies have demonstrated the central importance of glutathione in Nrf2-mediated neuroprotection [6], [27], [28]. However, the exact mechanisms of Nrf2-mediated protection in astrocytes has yet to be identified. In light of this, we have undertaken the current study using a combination of quantitative proteomics and siRNA screening to elucidate the specific mechanisms of astrocytic Nrf2 protection against oxidative stress.

## Results

### Nrf2 Activation Protects Astrocytes from Hydrogen Peroxide

**Figure 1:**
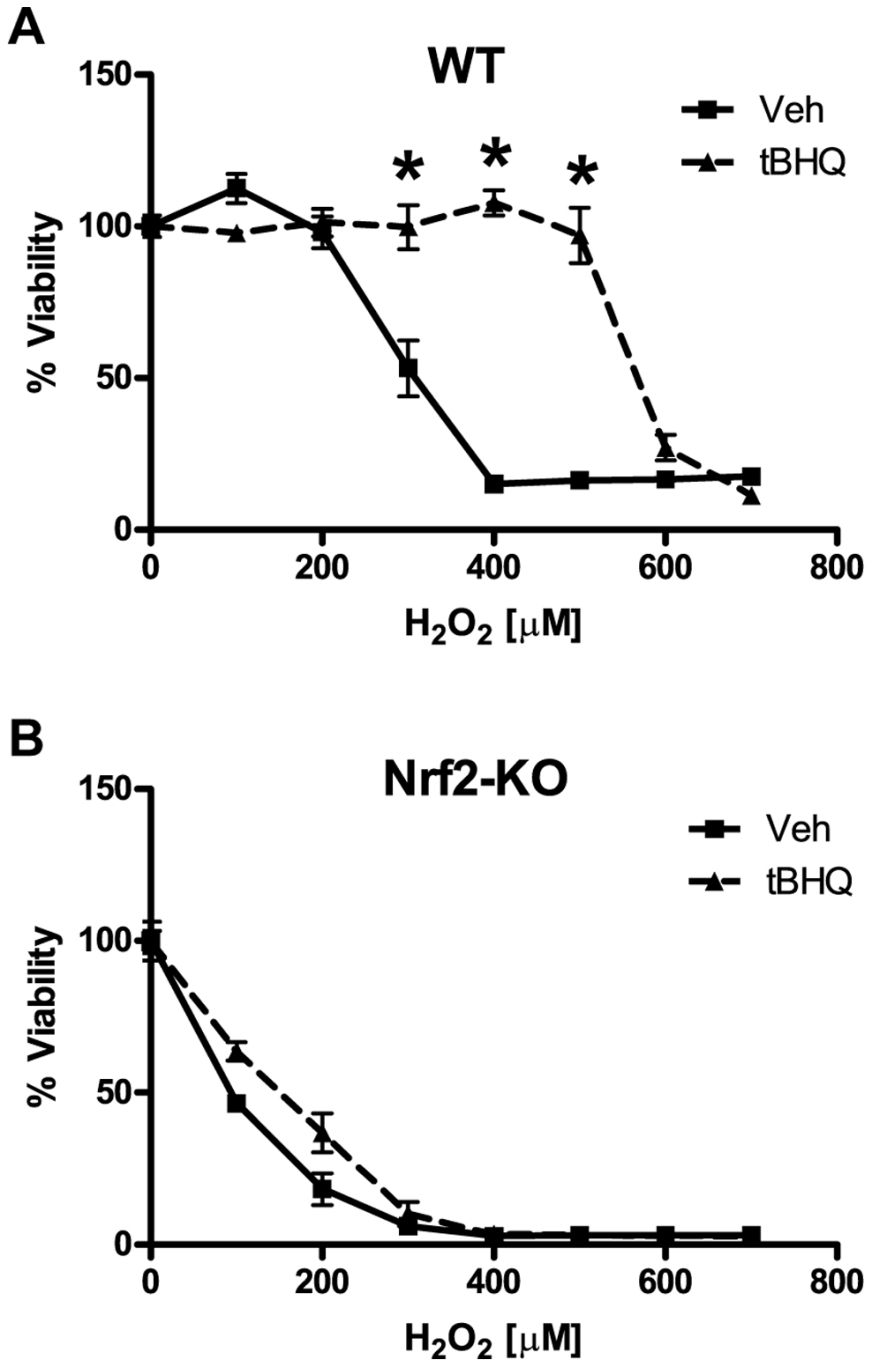
Effects of Nrf2 induction on H_2_O_2_ toxicity. **A)** Wild-type (WT) or **B)** Nrf2-knockout (Nrf2-KO) astrocytes were pretreated with vehicle or tBHQ and then treated with H_2_O_2_ as indicated. Cell viability was determined by MTS. Statistics were performed using 2-way ANOVA, * indicates p<0.01.

Although Nrf2 induction has previously been shown to protect primary astrocyte cultures from H_2_O_2_ toxicity, we sought to establish a toxicity assay to identify specific mechanisms of Nrf2 protection [5], [6], [7]. In order to achieve this, we generated H_2_O_2_ toxicity curves and then assessed cell viability by 3-(4,5-dimethylthiazol-2-yl)-5-(3-carboxymethoxyphenyl)-2-(4-sulfophenyl)-2H tetrazolium (MTS) and lactate dehydrogenase release (LDH) assays. Nrf2 induction with tBHQ confers robust protection against H_2_O_2_ toxicity (**Figure 1A**). Furthermore, this protection is reversed in Nrf2 knockout astrocytes, demonstrating that Nrf2 is required for tBHQ protection (**Figure 1B**). In addition to tBHQ treatment, adenoviral overexpression of Nrf2 was also able to confer robust protection (**Figure S1A**). To validate the results of the MTS assay, we also assessed toxicity by LDH assay, the results of which closely mirror that of the MTS assay (**Figure S1B**). These data show a robust Nrf2-dependent protection against H_2_O_2_ toxicity in primary astrocytes.

### Nrf2 Reduces ROS Induced by H_2_O_2_ Treatment and Enhances H_2_O_2_ Clearance

**Figure 2:**
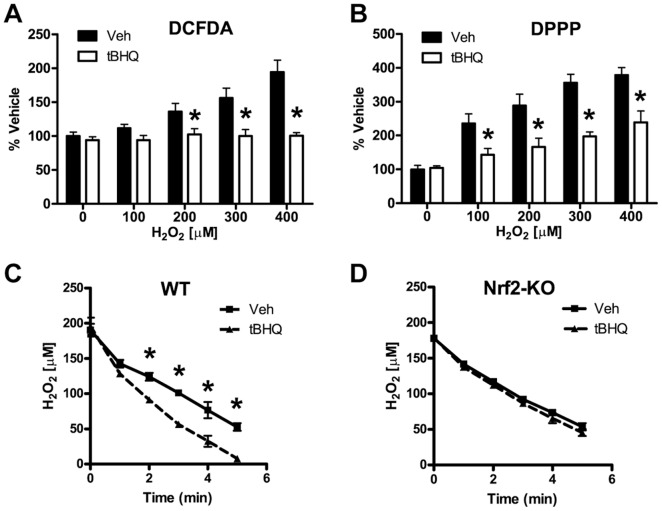
Effects of Nrf2 induction on ROS generation and extracellular H_2_O_2_ clearance. Wild-type astrocytes were pretreated with vehicle or tBHQ. H_2_O_2_ was added as indicated and after 4 hours the generation of ROS was monitored by either **A)** DCFDA or **B)** DPPP. Astrocytes were pretreated with vehicle or tBHQ and the rate of H_2_O_2_ clearance from the extracellular medium was measured over time for **C)** Wild-type (WT) or **D)** Nrf2-knockout (Nrf2-KO) cells. Statistics were performed using 2-way ANOVA, * indicates p<0.01.

We hypothesized that Nrf2 protects astrocytes from H_2_O_2_ toxicity by reducing the levels of reactive oxidative species (ROS) produced during H_2_O_2_ treatment. In order to assess this hypothesis, we monitored ROS levels by 2′,7′-dichlorodihydrofluorescein diacetate (DCFDA), which is a general ROS indicator, and diphenyl-1-prenylphosphine (DPPP), which is an indicator of lipid oxidation [29]. In vehicle treated astrocytes, the levels of ROS (as indicated by DCFDA fluorescence) and lipid oxidation (as indicated by DPPP fluorescence) increased with higher concentrations of H_2_O_2_ (**Figure 2A, B**). Nrf2 induction reduced levels of ROS and lipid oxidation in comparison to vehicle treatment (**Figure 2A, B**). This effect was particularly dramatic at the highest concentrations of H_2_O_2_. To further investigate how Nrf2 activation reduces the levels of ROS, we assessed the effect of Nrf2 activation on the clearance of extracellular H_2_O_2_. We added H_2_O_2_ directly to primary astrocyte cultures and then monitored the clearance of H_2_O_2_ from the culture medium over time. As has been previously shown, astrocytes exhibit a robust capacity to clear extracellular hydrogen peroxide [30], [31], [32], [33]. In wild-type astrocytes, Nrf2 activation resulted in an almost 2-fold increase in H_2_O_2_ clearance over the vehicle treated cells (**Figure 2C** **and** **Table 1**). In Nrf2 knockout (Nrf2-KO) astrocytes, the H_2_O_2_ clearance rate was unchanged after tBHQ treatment, demonstrating the dependence of this effect on Nrf2 (**Figure 2D** **and** **Table 1**). As these data demonstrate, Nrf2 activation significantly enhances the antioxidant capacity of astrocytes, resulting in a more robust clearance of H_2_O_2_ and a reduction of both the ROS and lipid oxidation levels produced by H_2_O_2_ treatment.

**Table 1:** Extracellular Peroxide Clearance.

		1/2 Time min	Clearance µmole/(min×mg)	Clearance % Veh
WT	Veh	3.3	4.8	
	tBHQ	1.7	9.5	190
Nrf2-KO	Veh	3.0	5.4	
	tBHQ	2.6	6.2	113

### Nrf2 Protection against tBOOH and 4-HNE

**Figure 3:**
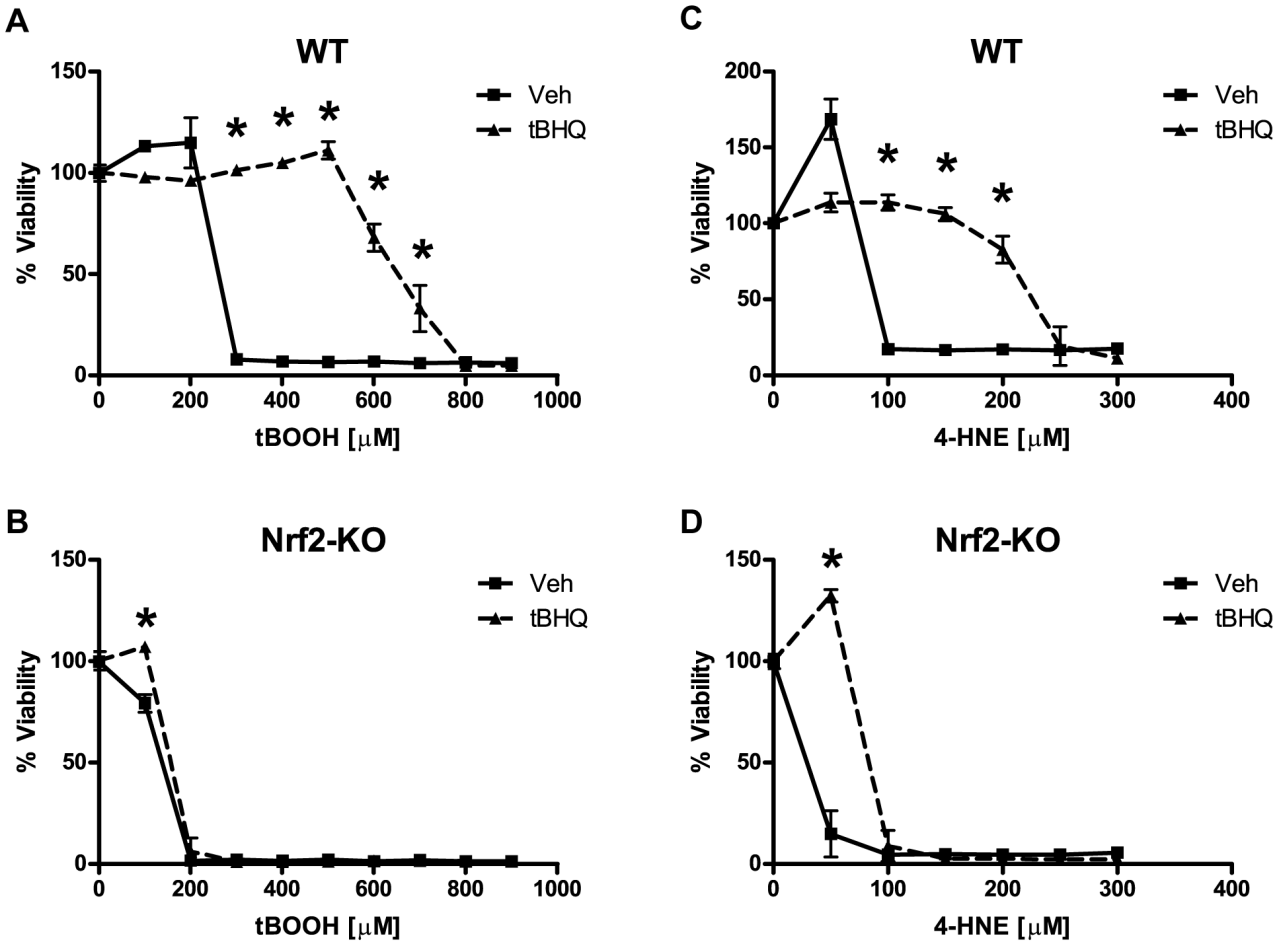
Effects of Nrf2 induction on tBOOH and 4-HNE toxicity. Wild-type (WT) or Nrf2-knockout (Nrf2-KO) astrocytes were pretreated with vehicle or tBHQ (40 µM) for 48 hours and then treated with tBOOH, **A)** WT and **B)** Nrf2-KO, or with 4-HNE, **C)** WT and **D)** Nrf2-KO. Cell viability was determined by MTS. Statistics were performed using 2-way ANOVA, * indicates p<0.01.

To examine the role of the Nrf2-ARE pathway in protecting astrocytes from other oxidative stressors, we chose the organic peroxide *tert*-butyl hydroperoxide (tBOOH) and 4-hydroxynonenal (4-HNE) both of which are produced during conditions of oxidative stress. Organic peroxides, such as tBOOH, are produced physiologically during eicosanoid metabolism. Lipid peroxidation products, such as 4-HNE, are produced after the reaction of free radicals with cellular lipids [1]. Nrf2 activation via tBHQ produced robust protection against both tBOOH and 4-HNE (**Figure 3A, B**). This protection was reversed in Nrf2-KO astrocytes, demonstrating the requirement of Nrf2 for tBHQ protection (**Figure 3C, D**).

### Identification of Nrf2 Regulated Proteins by Quantitative Proteomics

**Figure 4:**
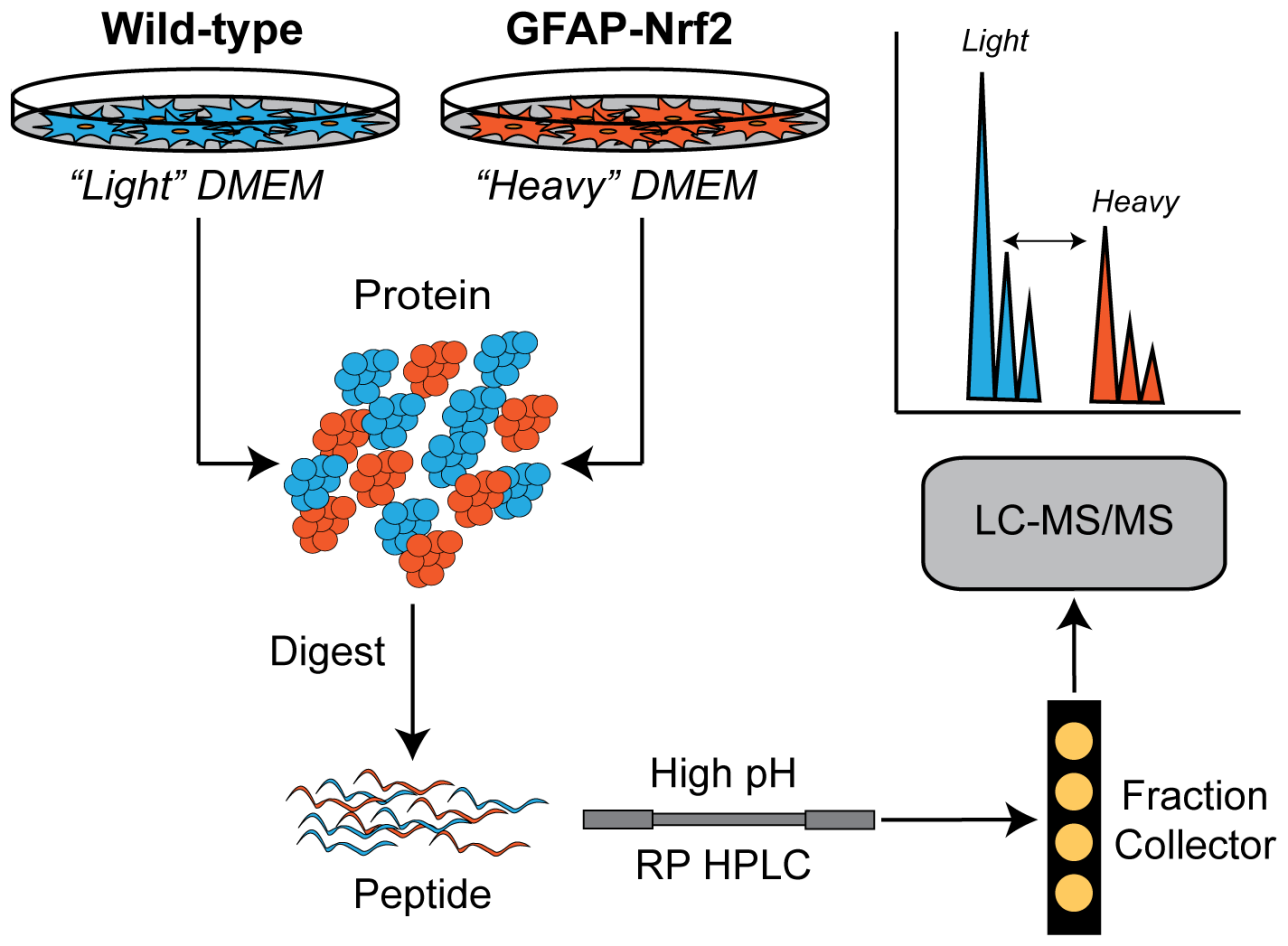
Overview of SILAC workflow. Astrocytes were grown in “light” or “heavy” amino acid containing media. Proteins from the wild-type (“light” labeled) and GFAP-Nrf2 (“heavy” labeled) cells were mixed and digested using trypsin. Tryptic peptides were then separated offline by high pH reverse phase HPLC. Fractions were collected and then individually analyzed by LC-MS/MS. The differences in expression were quantified by calculating the area under the curve for the “light” and “heavy” peptide pairs for each protein.

In order to identify protein targets of the Nrf2-ARE pathway, we performed quantitative proteomics on primary astrocytes isolated from mice that overexpress Nrf2 under the control of the astrocyte-specific promoter glial fibrillary acid protein (GFAP-Nrf2) [26]. We used stable isotope labeling by amino acids in cell culture (SILAC) and quantitative shotgun proteomics to quantify changes in protein expression between wild-type and GFAP-Nrf2 astrocytes (see **Figure 4** for an overview). We filtered the results of these experiments based on a fold-change cutoff and a requirement that a protein be identified and quantified in all three experimental replicates. From these experiments, we identified 72 differentially regulated proteins (**Table S3**). In order to further validate the proteomics results and distill our target genes/proteins into an experimentally manageable number, we compared the list of differentially expressed proteins with differentially regulated mRNA transcripts that were previously identified by microarray in our laboratory [5], [7]. After filtering each microarray dataset according to a relative fold-change and rank analysis threshold, we compared the identified mRNA transcripts against the proteins identified by quantitative proteomics. We required each putative Nrf2-regulated gene/protein to be differentially regulated in at least two out of the three datasets. From this analysis, we identified 29 differentially regulated genes/proteins (**Table 2**). In order to identify those enzymes that might be critical for the antioxidant and protective capacities of the Nrf2-ARE pathway, we performed a gene ontology functional annotation analysis using the DAVID Bioinformatic Resources. According to this analysis, a number of functional categories related to oxidative stress and/or cellular redox status were significantly enriched (**Table S4)**. We combined those genes/proteins identified in the top three gene ontology terms related to oxidative stress (GO:0006979, 0055114, and 0045454) to generate a target list of 15 genes/proteins, including genes associated with the production and utilization of NADPH and glutathione as well as enzymes associated with the direct enzymatic detoxification of reactive oxygen species (**Table S5**). After an extensive review of the literature, we further refined our target list to a core set of eight enzymes that we hypothesized might be necessary for Nrf2-mediated protection against oxidative stressors, including: NAD(P)H dehydrogenase (quinone)-1 (NQO1), heme oxygenase-1 (HO-1), catalase (CAT), glutathione S-transferase A4 (GSTA4), prostaglandin reductase-1 (PTGR1), glutamate-cysteine ligase modifier subunit (GCLM), and peroxiredoxin-1 and -6 (Prdx1 and Prdx6).

**Table 2:** Comparison of Quantitative Proteomics and Microarray Datasets.

SwissProt ID	DAVID Description	SILAC	Microarray 1	Microrray 2
O08709	peroxiredoxin 6	2.11	1.70	2.43
O08739	adenosine monophosphate deaminase 3	NA	2.33	2.14
O54754	aldehyde oxidase 1	NA	2.62	7.30
P06801	malic enzyme 1	NA	1.74	3.45
P10649	glutathione S-transferase, mu 1	2.14	1.54	2.19
P14901	heme oxygenase (decycling) 1	10.41	2.65	NA
P17809	solute carrier family 2, member 1	NA	1.49	5.15
P19639	glutathione S-transferase, mu 3	NA	1.77	1.67
P24270	catalase	NA	1.93	1.61
P24472	glutathione S-transferase, alpha 4	4.85	3.95	9.53
P35700	peroxiredoxin 1	2.74	1.60	1.68
P19157	glutathione S-transferase, pi 1	2.71	2.34	2.72
P47791	glutathione reductase	14.95	2.60	NA
P52760	heat-responsive protein 12	3.01	2.58	4.88
P56395	cytochrome b-5	NA	1.63	2.10
P97494	glutamate-cysteine ligase, catalytic subunit	NA	2.24	1.57
Q00612	glucose-6-phosphate dehydrogenase	NA	2.15	3.24
Q60963	phospholipase A2, group VII	NA	2.70	2.44
Q64337	p62	NA	1.61	1.72
Q64669	NAD(P)H dehydrogenase, quinone 1	3.80	5.10	NA
Q91YR9	prostaglandin reductase 1	NA	2.94	4.30
Q93092	transaldolase 1	2.42	2.94	3.32
Q9D379	epoxide hydrolase 1, microsomal	NA	1.98	3.32
Q9D6Y9	glucan (1,4-alpha-), branching enzyme 1	NA	2.01	22.51
Q9D975	sulfiredoxin 1	NA	10.07	10.73
Q9DCD0	phosphogluconate dehydrogenase	3.54	1.97	2.01
Q9JMH6	thioredoxin reductase 1	2.77	3.87	2.93
Q9R0P3	esterase D/formylglutathione hydrolase	NA	2.42	2.34
Q9R0P9	ubiquitin carboxy-terminal hydrolase L1	1.50	1.48	NA

### Nrf2 Modulated Genes

**Figure 5:**
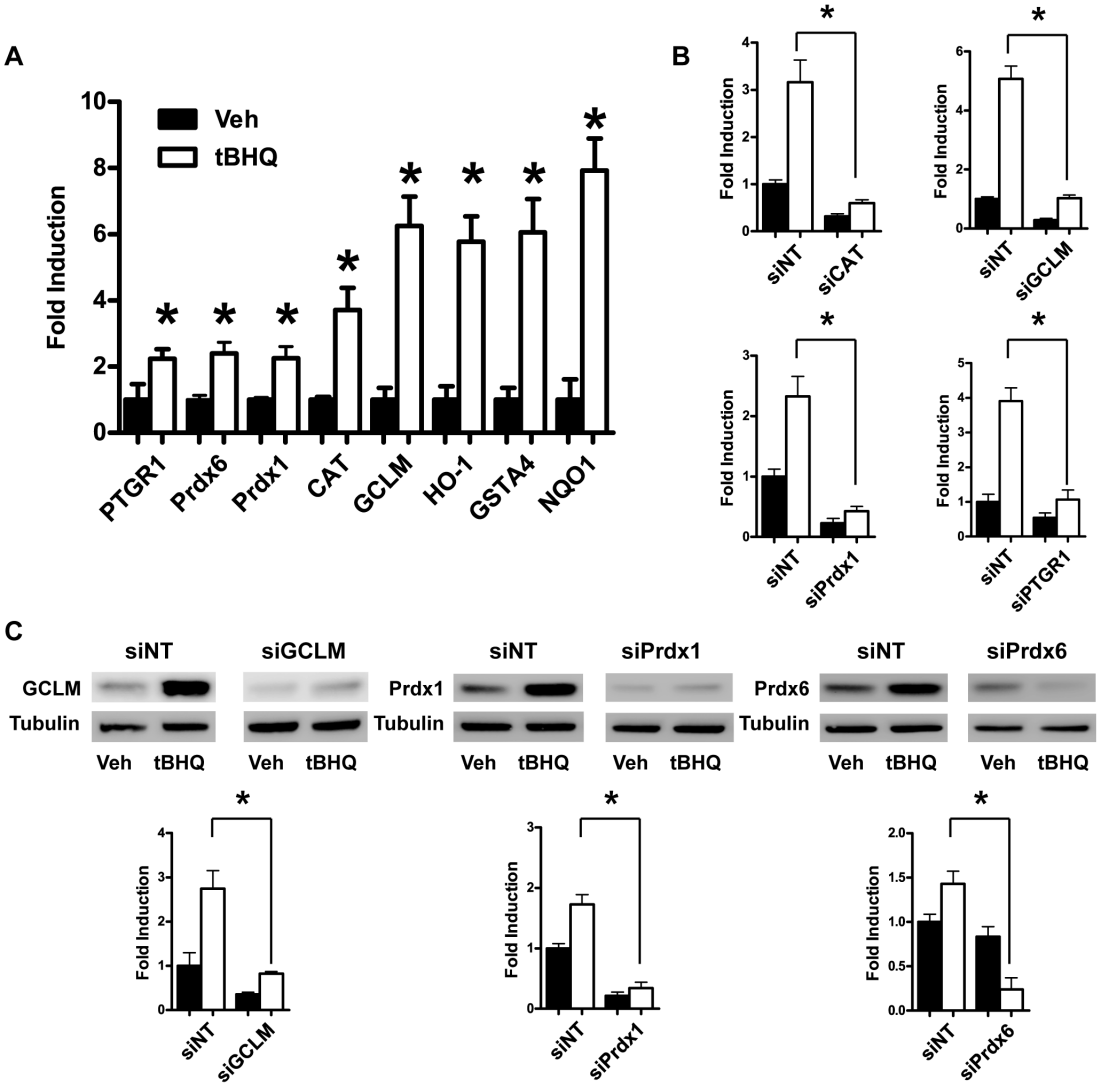
Validation of Nrf2 targets and siRNA knockdown by quantitative PCR and western blot. **A)** Wild-type (WT) astrocytes were treated with vehicle or tBHQ. Differences in target gene expression were analyzed by qPCR. Statistics were performed on vehicle versus tBHQ for each gene using a paired t-test, * indicates p<0.05. WT astrocytes were pretreated with siRNA constructs against the indicated genes and a non-targeting siRNA control (siNT) prior to vehicle or tBHQ treatment. RNA or protein extracts were subjected to **B)** qPCR and/or **C)** western blot to validate knockdown. Statistics were performed on siNT plus tBHQ versus targeted siRNA plus tBHQ for each gene or protein using a paired t-test, * indicates p<0.05.

In order to examine transcript level changes of our core enzymes, we performed quantitative PCR (qPCR). All of eight target genes were robustly activated by tBHQ treatment (**Figure 5A**). In addition, the mRNA levels of these same genes were strongly upregulated in astrocytes infected with an Nrf2 adenoviral construct as determined by qPCR (data not shown). To identify the contribution of these individual genes to the Nrf2 protection, we used siRNA constructs to knockdown each of the eight genes. The siRNA knockdown was validated by qPCR and/or western blot (**Figure 5B, C**).

### The Role of Catalase in Nrf2 Protection against H_2_O_2_

**Figure 6:**
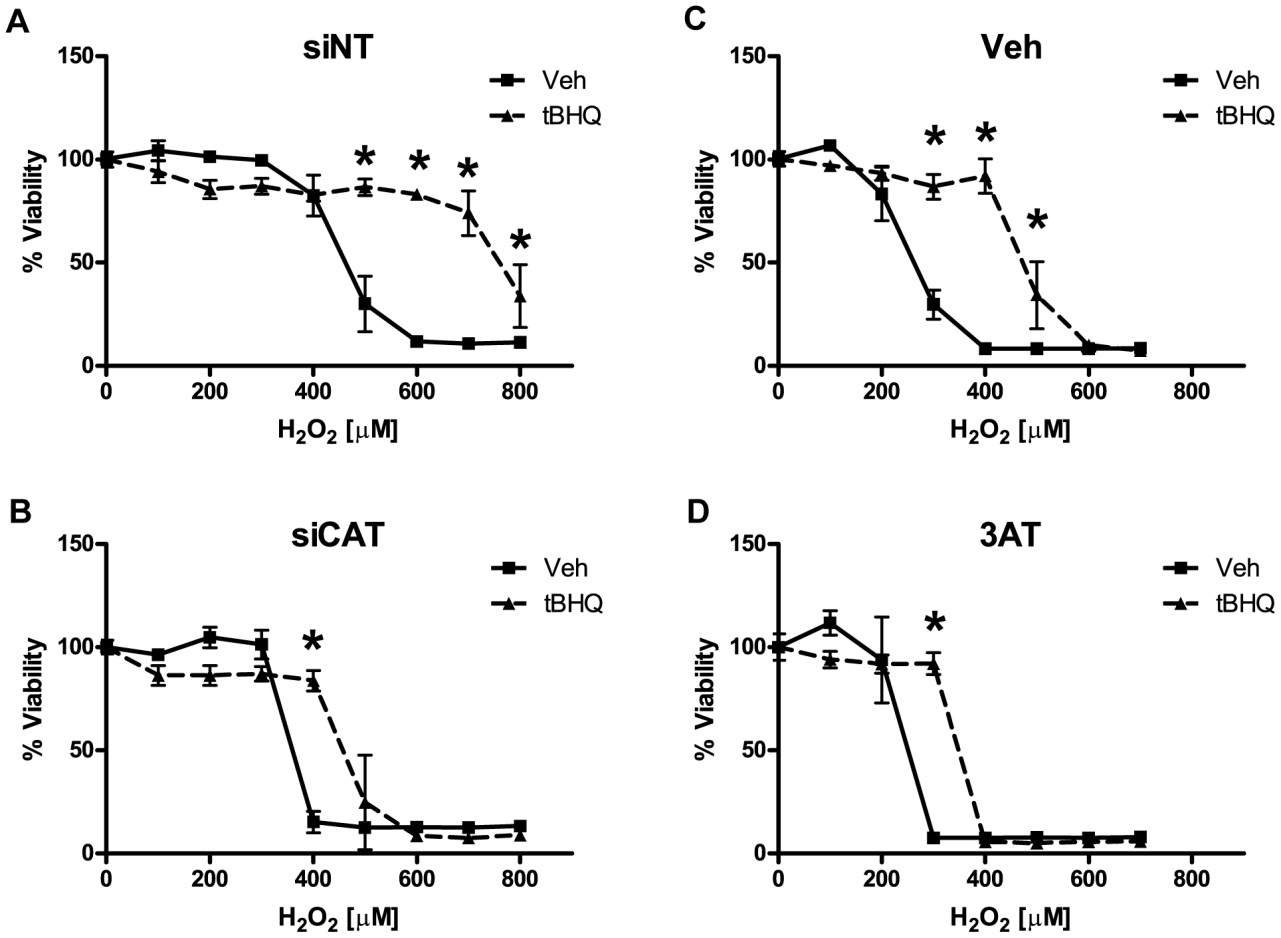
The effect of catalase siRNA knockdown on Nrf2 protection against H_2_O_2_. Vehicle or tBHQ treated astrocytes were pretreated with **A)** non-targeting siRNA (siNT) or **B)** catalase siRNA (siCAT) or **C)** a vehicle control (Veh) or **D)** a catalase inhibitor, 3-aminotriazole (3AT). H_2_O_2_ toxicity curves were performed as indicated. Cell viability was determined by MTS. Statistics were performed using 2-way ANOVA, * indicates p<0.01.

After the knockdown of all each individual gene by siRNA, astrocytes were treated with tBHQ to activate Nrf2 and then treated with H_2_O_2_ to assess the effects of siRNA knockdown on Nrf2-mediated protection. The relative protection afforded by tBHQ treatment was robustly reduced in the cells treated with siRNA against catalase (siCAT) when compared to the non-targeting siRNA (siNT) treated cells (**Figure 6A, C**). To validate the siRNA data, we treated astrocytes with the specific catalase inhibitor 3-aminotriazole (3AT). Chemical inhibition by 3AT also reversed Nrf2 protection to a similar degree as siRNA treatment (**Figure 6B, D**). Knockdown of other candidate genes did not substantially modulate Nrf2 protection against H_2_O_2_ (data not shown).

### The Role of Prdx6 in Nrf2 Protection against tBOOH

**Figure 7:**
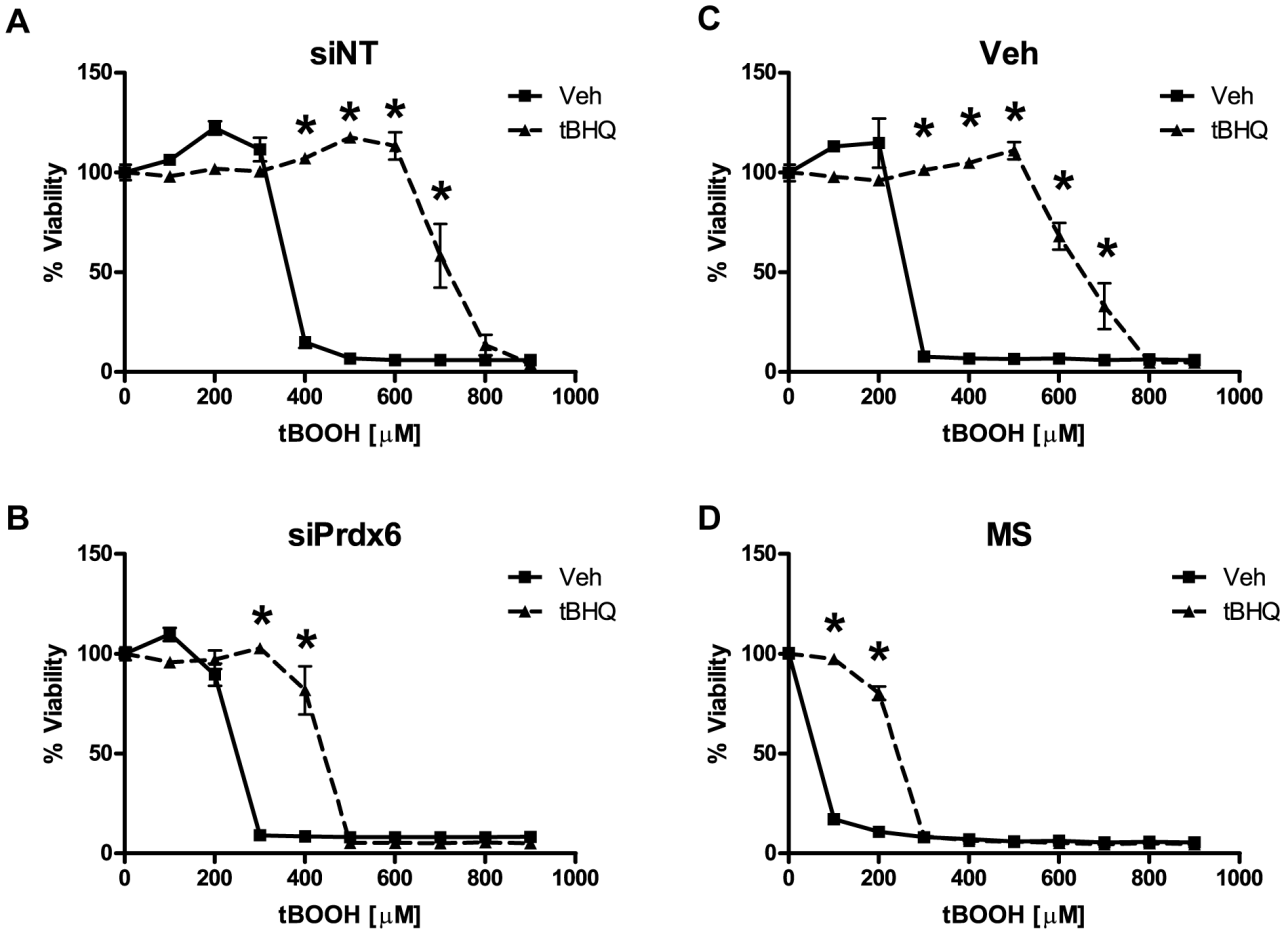
The effect of Prdx6 siRNA knockdown on Nrf2 protection against tBOOH. Vehicle or tBHQ treated astrocytes were pretreated with **A)** non-targeting siRNA (siNT) or **B)** peroxiredoxin-6 siRNA (siPrdx6) or with either **C)** a vehicle control (Veh) or **D)** a peroxiredoxin-6 inhibitor, mercaptosuccinate (MS). tBOOH toxicity curves were performed as indicated. Cell viability was determined by MTS. Statistics were performed using 2-way ANOVA, * indicates p<0.01.

To evaluate the relative contribution of individual genes to Nrf2 protection against tBOOH, we used siRNA constructs to knockdown each candidate gene before treatment with tBOOH. Nrf2 protection was considerably reduced after knockdown of Prdx6 (**Figure 7A, C**). In order to validate the siRNA data, we treated astrocytes with the Prdx6 inhibitor mercaptosuccinate (MS) before treatment with tBOOH. In concordance with the siRNA data, mercaptosuccinate reversed the Nrf2-mediated protection against tBOOH (**Figure 7B, D**). Knockdown of other candidate genes did not substantially modulate Nrf2 protection against tBOOH (data not shown).

### The Role of PTGR-1 in Nrf2 Protection against 4-Hydroxynonenal

**Figure 8:**
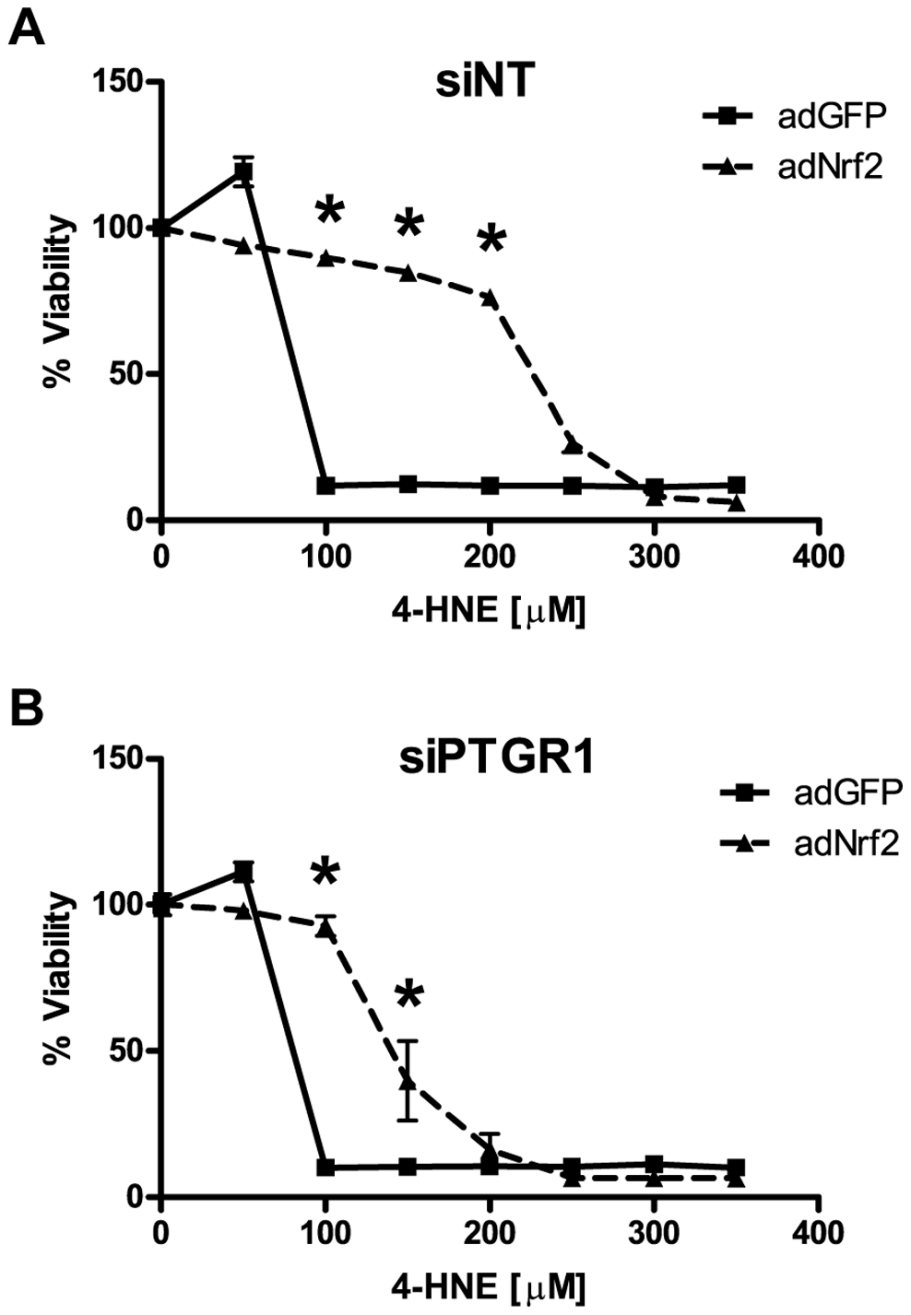
The effect of PTGR1 siRNA knockdown on Nrf2 protection against 4-HNE. Vehicle or tBHQ treated astrocytes were pretreated with **A)** non-targeting siRNA (siNT) or **B)** prostaglandin reductase-1 siRNA (siPTGR1). 4-HNE toxicity curves were performed as indicated. Cell viability was determined by MTS. Statistics were performed using 2-way ANOVA, * indicates p<0.01.

In addition to modulating enzymes directly involved in the processing of ROS, Nrf2 also modulates the expression of genes responsible for the detoxification of lipid oxidation products, including glutathione S-transferase-A4 (GSTA4) and prostaglandin reductase-1 (PTGR1). In order to examine the role of individual enzymes in Nrf2 protection against 4-HNE, we used siRNA to knockdown each candidate gene before treatment with 4-HNE. The knockdown of PTGR-1 considerably reduced Nrf2-mediated protection against 4-HNE (**Figure 8A, B**). Knockdown of the other candidate genes did not substantially modulate Nrf2 protection against 4-HNE (data not shown).

### Nrf2 Protection and Glutathione

**Figure 9:**
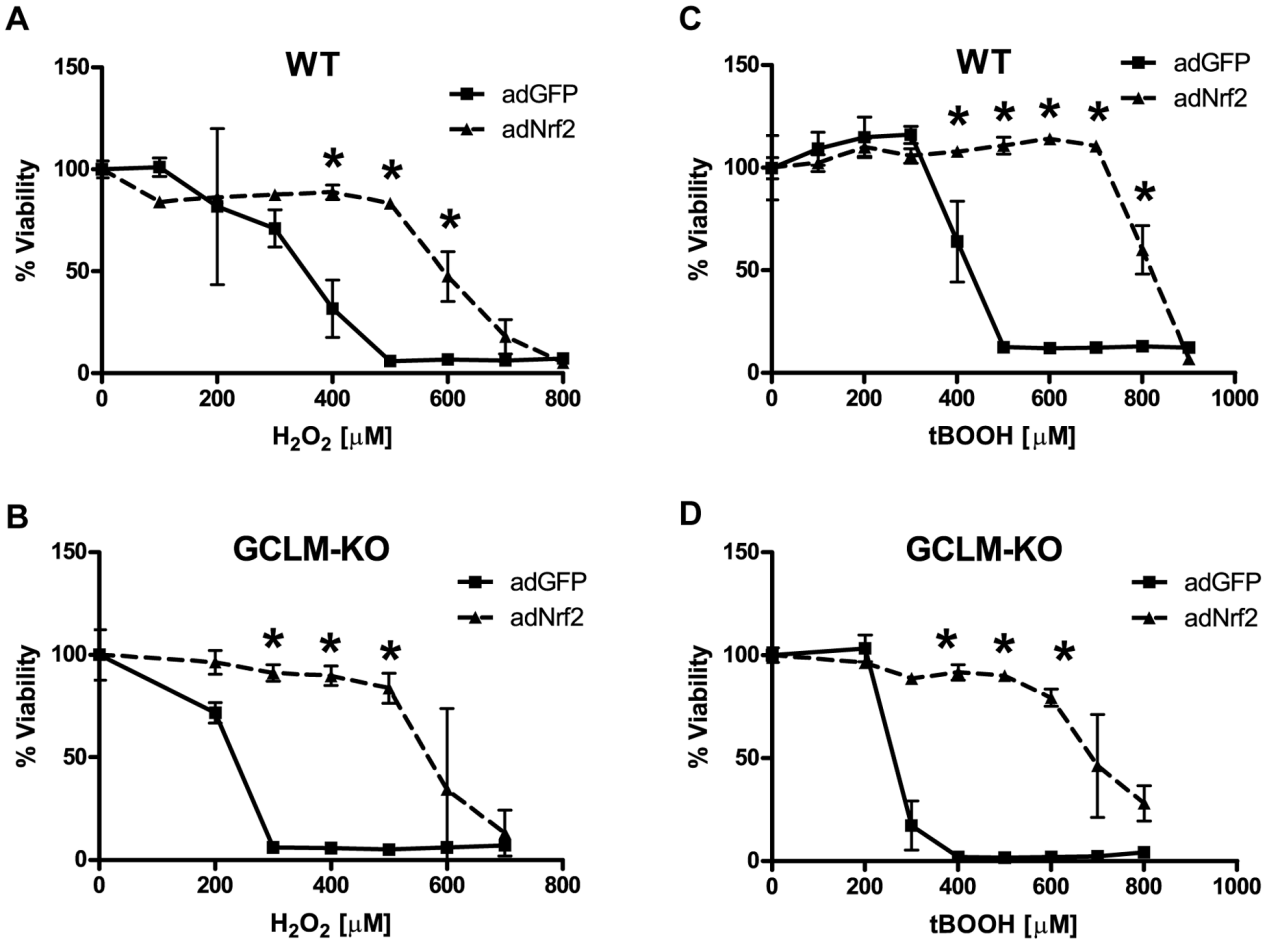
The effects of glutathione deficiency on Nrf2 protection. Wild-type (WT) or glutamate-cysteine ligase, modifier subunit knockout (GCLM-KO) astrocytes were infected with adGFP or adNrf2 virus prior to stressor treatment. **A)** WT cells or **B)** GLCM-KO cells were treated with H_2_O_2_ as indicated. **C)** WT cells or **D)** GLCM-KO cells were treated with tBOOH as indicated. Cell viability was determined by MTS. Statistics were performed using 2-way ANOVA, * indicates p<0.01.

In order to evaluate the contribution of glutathione to the protective effects of Nrf2, we induced Nrf2 expression in primary astrocytes isolated from mice that lack the glutamate-cysteine ligase modifier subunit (GCLM-KO) and then treated them with H_2_O_2_ or tBOOH [34]. Nrf2 activation conferred robust protection against both H_2_O_2_ and tBOOH in GCLM-KO astrocytes (**Figure 9C, D**). In addition, siRNA knockdown of GCLM had no effect on Nrf2 protection against 4-HNE (**Figure S2**).

## Discussion

In order to elucidate mechanisms of astrocytic Nrf2 protection, we used molecular, biochemical, and proteomics approaches to identify specific enzymes responsible for Nrf2 protection against oxidative stress. One of the oxidative stressors used in this study, H_2_O_2_, is relatively unreactive towards biomolecules; however, it rapidly reacts with physiological iron via the Fenton reaction to produce the highly reactive and damaging hydroxyl radical [1]. The hydroxyl radical can damage DNA, protein, lipids, and organelles. In order to examine changes in ROS and lipid oxidation as a result of H_2_O_2_ treatment, we employed the fluorescent ROS probes DCFDA and DPPP (**Figure 2A, B**). These assays were performed 4 hours after H_2_O_2_ treatment and were an assessment of changes in ROS and lipid oxidation levels which occur as a secondary consequence of H_2_O_2_ treatment [30], [31], [32], [33]. This secondary production of ROS and lipid oxidation is most likely due to damage to the mitochondrial electron transport chain and/or the induction of NADPH oxidases. H_2_O_2_ has been shown to damage mitochondrial DNA and lipids as well as disrupt the electron transport chain, causing an increase in mitochondrial superoxide production [35], [36]. In addition, recent evidence has emerged implicating H_2_O_2_ as a signaling molecule capable of stimulating ROS production via NADPH oxidases [37], [38]. In this study, Nrf2 induction produced a robust increase in cellular antioxidant capacity that reduced ROS and lipid oxidation as well as enhanced extracellular H_2_O_2_ clearance (**Figure 2A, B**).

In order to identify the Nrf2-regulated enzymes responsible for these changes, we used quantitative proteomics in combination with stable isotope labeling by amino acids in cell culture (SILAC) to identify differentially expressed proteins in GFAP-Nrf2 astrocytes (**Figure 4**). Typically in a SILAC experiment, cells from one experimental condition are grown in a medium that contains stable isotopologues of essential amino acids while cells from the other condition are grown in a medium with natural amino acids. The amino acids from the medium are incorporated into the cellular proteome as the cells grow and proliferate, producing differentially labeled “light” and “heavy” protein samples. Protein extracts from the two experimental samples are then harvested, mixed, digested, and analyzed by mass spectrometry (MS). The relative protein expression changes between the two samples are then quantified by comparing relative MS signal intensity between the “light” and “heavy” peptide peaks. Using this technique, we were able to identify 72 differentially expressed proteins in GFAP-Nrf2 versus wild-type (WT) astrocytes–25 proteins were increased and 47 proteins were decreased relative to the WT control (**Table S3**).

In order to identify genes/proteins that are important for Nrf2-mediated protection, we used an iterative approach to refine our dataset. First, we compared the proteins identified in the proteomics screen to two previously published mRNA microarray data sets [5], [7]. By doing this we significantly reduced the number of putative Nrf2-regulated genes to a total of 29 (**Table 2**). There is a high degree of concordance between the microarray and proteomics datasets. However, the proteomics analysis also revealed an additional 11 upregulated proteins that were not identified in the microarray datasets (**Table S3**). It is interesting to note that over a third of these proteins are ubiquitin-conjugating enzymes, indicating the possible importance of the Nrf2 pathway in the regulating ubiquitin conjugation under conditions of oxidative stress. Kopito and co-workers have shown the importance of Nrf2 in the ubiquitin-autophagy pathway via its regulation of p62 [39]. There were no commonly downregulated genes among the datasets (data not shown). It is clear that analyzing both microarray and proteomics data in parallel is a powerful approach that can reduce spurious results as well as reveal subtle differences in transcript- and protein-level regulation of protein expression.

From these data, catalase was identified as a potentially important contributor to Nrf2-mediated protection against ROS. Catalase is one of the earliest antioxidant enzymes discovered and plays a central role in cellular protection against ROS by catalyzing the decomposition of H_2_O_2_ into water and oxygen [40]. The importance of catalase for the clearance and protection of astrocytes from H_2_O_2_ toxicity is well established [31], [41], [42], [43]. However, the importance of catalase in Nrf2-mediated protection in astrocytes was not known before this study. After siRNA knockdown or chemical inhibition of catalase, Nrf2 protection against H_2_O_2_ was almost completely ablated (**Figure 6**). These data indicate the central importance of catalase in Nrf2-mediated protection against H_2_O_2_.

Two enzymes that are thought to be important in oxidative signaling, peroxiredoxin-1 (Prdx1) and peroxiredoxin-6 (Prdx6), were both strongly induced by Nrf2 (**Figure 5A**). There are six known mammalian members of the peroxiredoxin family. Peroxiredoxin-1 through -5 are thioredoxin-dependent enzymes while Prdx6 is glutathione-dependent [44]. Peroxiredoxins are primarily known as peroxidases; however, they possess important functions that are distinct from their peroxidase activity [45], [46], [47], [48]. In the case of Prdx6, the oxidation of the specific reactive cysteines changes it from a peroxidase to a phospholipase [46]. In the case of Prdx1, cysteine oxidation results in the oligomerization of Prdx1 into a protein chaperone [45]. These changes are thought to be important in modulating oxidative signaling and inducing cell survival pathways [48].

In regards to Nrf2 protection, Prdx6 appears to play a substantial role in Nrf2 protection against tBOOH. Prdx6 knockdown by siRNA and chemical inhibition by mercaptosuccinate (MS) dramatically reduced Nrf2 protection against tBOOH (**Figure 7**). While Prdx6 is catalytically less efficient than glutathione peroxidase, it is significantly more important in protecting cells against tBOOH than glutathione peroxidase-1 [49]. Additionally, Prdx6 is upregulated in patients with Alzheimer’s disease and ALS, suggesting a potential role in neurodegenerative disease [50], [51], [52].

Sulfiredoxin is another target of Nrf2 and maintains Prdx1 activity by preventing its catalytic cysteine from becoming hyperoxidized. Sulfiredoxin has also been shown to be protective against oxidative stress [39]. However, in the context of the experiments presented here, knockdown by siRNA produced an increase of both basal resistance and Nrf2-mediated protection against H_2_O_2_ (**Figure S3**). We have no explanation for this unexpected result. However, it is clear that sulfiredoxin is vitally important to redox signaling and cellular responses to oxidative stress and its regulation by Nrf2, as well as that of Prdx1 and Prdx6, could represent a central control point for redox signaling via the Nrf2-ARE pathway.

One of the most studied toxic byproducts of lipid oxidation is 4-hydroxynonenal (4-HNE). 4-HNE is formed when polyunsaturated fatty acids undergo free radical reactions during conditions of oxidative stress. 4-HNE readily reacts with lysine, cysteine, and histidine to form protein adducts [53]. These 4-HNE adducts have been implicated in the etiology of Alzheimer’s disease and Parkinson’s disease [54], [55]. Nrf2 activation robustly protects astrocytes from 4-HNE toxicity (**Figure 3**) and regulates genes responsible for detoxifying 4-HNE, including both glutathione *S*-transferase A4 (GSTA4) and prostaglandin reductase-1 (PTGR1) (**Figure 5**) [5], [7], [56], [57], [58]. GSTA4 detoxifies 4-HNE via direct conjugation while PTGR1 catalyzes the reduction of the highly reactive *α,β*-carbon double bond of 4-HNE to a non-reactive single carbon bond [58]. The knockdown of PTGR1 had a dramatic effect on the ability of Nrf2 to protect against 4-HNE toxicity (**Figure 8**). However, siRNA knockdown of GSTA4 had no effect on Nrf2-mediated resistance to 4-HNE (data not shown). These data indicate the importance of PTGR1 in Nrf2 protection against 4-HNE.

In astrocytes, Nrf2 has been shown to modulate enzymes responsible for the production and processing of glutathione as well as enzymes which utilize glutathione for cellular detoxification processes [5], [6], [7], [27]. A number of studies have demonstrated the importance of glutathione in Nrf2-mediated neuroprotection [6], [27], [28]. The rate-limiting enzyme in the glutathione biosynthetic pathway is γ-glutamylcysteine ligase (GCL). This enzyme is composed of a catalytic subunit (GCLC) and a modifier subunit (GCLM). GCLM expression controls the rate of glutathione biosynthesis by modulating the catalytic efficiency of GCLC [34]. GCLM is highly induced by Nrf2 (**Figure 5**) and knockdown of GCLM by siRNA completely inhibits the ability of Nrf2 to increase total glutathione (data not shown). To study the role of glutathione in Nrf2 protection, we employed primary cortical astrocytes from GCLM knockout (GCLM-KO) mice that contain 80% less total glutathione than wild-type astrocytes. It has been shown that Nrf2 activation in GCLM-KO astrocytes fails to increase cellular levels of glutathione [59]. Surprisingly, we found that GCLM-KO astrocytes still exhibit a robust Nrf2 protection against H_2_O_2_ and tBOOH even without the ability to upregulate glutathione synthesis (**Figure 9**). In addition, siRNA knockdown of GCLM does not effect Nrf2 protection against 4-HNE (**Figure S2**). While these data are somewhat unexpected, other studies have shown that Nrf2 does not require *de novo* glutathione synthesis for its protective effects. For example, in mouse embryo fibroblasts isolated from GCLM-KO mice Nrf2 induction results in protection from arsenite, a compound known to induce ROS [60]. In addition, *in vivo* astrocyte-specific Nrf2 overexpression reversed many of the pathological hallmarks of Alexander’s disease while glutathione deficiency had no affect on pathology [24]. These data provide strong evidence that Nrf2 protection in astrocytes is largely independent of *de novo* glutathione synthesis.

Both NQO1 and HO-1 are widely recognized as canonical Nrf2 genes and both have been shown to possess potent cytoprotective activity. HO-1 catalyzes the degradation of heme to produce biliverdin that is subsequently converted to bilirubin, a powerful radical scavenger [61]. NQO1 has also been shown to be neuroprotective against oxidative damage by reducing highly reactive quinones to less reactive hydroquinones [62]. In the brain, HO-1 has been shown to be cytoprotective in models of stroke, excitotoxicity, Parkinson’s disease, and Alzheimer’s disease while NQO1 has been shown to be protective against a model of Parkinson’s disease [63], [64], [65], [66], [67], [68]. More recently, both HO-1 and NQO1 have been shown to be responsible for the neuroprotective properties of Nrf2 against H_2_O_2_ in astrocytes [69]. However, our data indicates no dependence of Nrf2 protection on either NQO1 or HO-1 for any of the stressors tested, including H_2_O_2_ (data not shown). It is unclear why our results differ from the study by Park and co-workers; however, in that study, chemical inhibitors of both HO-1 and NQO1 were used instead of the more specific siRNA knockdown used in our study.

**Figure 10:**
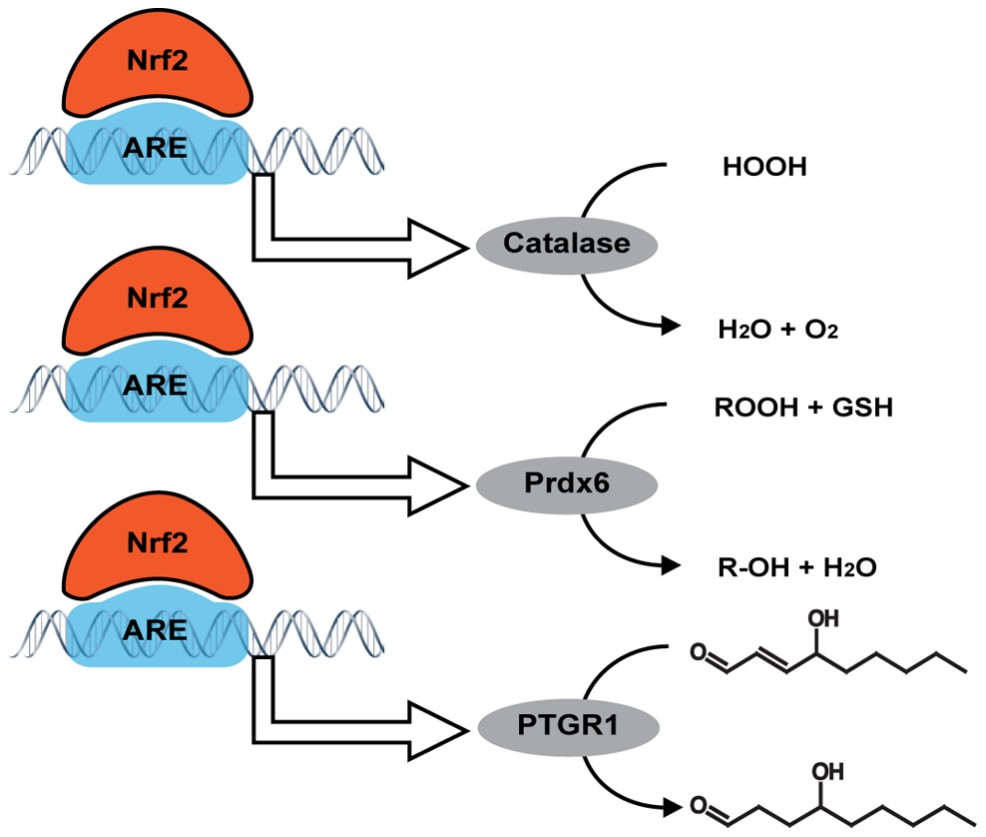
Overview of results. Binding of Nrf2 to the antioxidant response element (ARE) induces the expression of catalase, peroxiredoxin-6 (Prdx6), and prostaglandin reductase-1 (PTGR1). Each of these enzymes has a specific role in detoxifying the oxidative stressors H_2_O_2_, tBOOH, and 4-HNE.

In summary, we have shown the importance of the Nrf2-ARE pathway in protecting astrocytes against oxidative stress. This protection appears to require discrete enzymes working synergistically to detoxify specific oxidative stressors, i.e. catalase is required for Nrf2 protection against H_2_O_2_, Prdx6 is required for protection against tBOOH, and PTGR1 is required for protection against 4-HNE (summarized in **Figure 10**). Finally, while glutathione is certainly an extremely important physiological antioxidant and is essential for cellular survival, it does not appear to be critical for Nrf2 protection against the H_2_O_2_, tBOOH, or 4-HNE in astrocytes. These data indicate the complex mechanisms of Nrf2 protection and the requirement of multiple enzymes to execute the powerful protective effects of the Nrf2-ARE pathway.

## Materials and Methods

### Animals

Transgenic mice overexpressing Nrf2 under the astrocyte-specific glial fibrillary acid protein promoter (GFAP-Nrf2) and transgenic mice lacking the gene for glutamate-cysteine ligase modifier subunit (GCLM-KO) were utilized as indicated [26], [70]. This study was approved by the University of Wisconsin-Madison Institutional Animal Care and Use Committee (IACUC) and all animal procedures were performed in accordance with the requirements of the IACUC. The UW-Madison USDA Research Registration number is **35-R-1** and the Laboratory Animal Welfare (OLAW) Public Health Service (PHS) Assurance Number is **A3368-01**.

### Materials

The Hank’s balanced salt solution (HBSS), Dulbecco’s modified Eagle’s medium (DMEM), Opti-MEM, fetal bovine serum (FBS), penicillin/streptomycin, TRIzol, Lipofectamine, and 2′,7′-dichlorodihydrofluorescein diacetate (carboxy-H_2_DCFDA) were obtained from Life Technologies (Carlsbad, CA). 4-hydroxynonenal (4-HNE) and diphenyl-1-prenylphosphine (DPPP) were purchased from Cayman Chemical (Ann Arbor, MI). 3-(4,5-dimethylthiazol-2-yl)-5-(3-carboxymethoxyphenyl)-2-(4-sulfophenyl)-2H-tetrazolium (MTS), lactate dehydrogenase (LDH) release, and reverse transcriptase kits were purchased from Promega (Madison, WI). Heavy isotopes for SILAC labeling were purchased from Cambridge Isotopes (Andover, MA). All siRNA constructs and primers were obtained from Integrated DNA Technologies (Coralville, IA). Cycler480 SYBR Green I Master mix was purchased from Roche Applied Sciences (Indianapolis, IN). All other reagents were obtained from Sigma (St. Louis, MO).

### Primary Astrocyte Culture

Primary astrocyte cultures were prepared from postnatal day 1 (P1) mice. The cerebral cortices from individual P1 pups were removed, placed in ice-cold HBSS, minced with a scalpel blade, and then transferred to a tube with 10 ml of 0.25% trypsin at 37°C. After 25 minutes, 10 ml of DMEM+FBS (1.2 mg/ml sodium bicarbonate, 3.6 mg/ml HEPES, 10% fetal bovine serum (FBS), penicillin/streptomyocin at 100 IU/ml and 100 µg/ml) was added to deactivate the trypsin. DNase was added to a final concentration of 0.05 mg/ml and then the cells were pelleted at 400 *g* for 3 minutes. The supernatant was removed and the tissue pellet was re-suspended in 5 ml of fresh DMEM+FBS. After triturating 15–20 times with a 5 ml pipette, the disassociated cells passed through a 70 µm cell strainer. Cells from the cortices of three pups were plated onto a uncoated T75 flask. The culture medium was changed after the first day and then every 3 days thereafter. After the astrocytes reached confluency (7–8 days), the flasks were placed on a rotary shaker (200 rpm). After 18 hours, the cells were lifted with trypsin+EDTA and then re-plated at 35,000 cells/cm^2^. The astrocytes were allowed to reach confluency (5–7 days) before use. For the SILAC experiments, the DMEM was supplemented with either the natural isotopes of lysine and arginine for the “light” SILAC medium or “heavy” lysine (U-13C6, 99%; U-15N2, 99%, Cat# CNLM-291-H-0.1) and “heavy” arginine (U-13C6, 99%, Cat# M-2265-H-0.1) from Cambridge Isotopes (Andover, MA) for the “heavy” SILAC medium. Briefly, cells from the GFAP-Nrf2 pups were grown and maintained throughout the culturing period with “heavy” DMEM+FBS while the wild-type mice were grown and maintained with “light” DMEM+FBS. Please refer to **Figure 4** for an overview. Littermate controls were used for all experiments.

### Toxicity Curves

Astrocytes were treated with either adenovirus overexpressing GFP (control) and Nrf2 (50 MOI) or a vehicle control and a 40 µM *tert*-butyl hydroquinone (tBHQ), as previously published [7]. After 48 hours, toxicity curves were performed using hydrogen peroxide, *tert*-butyl-hydrogen peroxide, or 4-hydroxynonenal at the concentrations indicated. Each concentration of stressor was used to treat four replicates per condition. Additionally, 3-aminotriazol (a catalase inhibitor) at 10 mM, or mercaptosuccinate (a peroxiredoxin-6 inhibitor) at 0.5 mM, was added to cell cultures as indicated two hours prior to treatment. After 24 hours of treatment, cell viability was measured by either MTS or LDH assay, according to the manufacturer’s instructions. Viability was reported as a percentage of the untreated vehicle control.

### Measurement of ROS

DPPP (100 µM) or carboxy-H_2_DCFDA (10 µM) in DMEM without FBS was added to confluent astrocytes in a 96-well plate. After 30 minutes, the solution was replaced with DMEM+FBS without phenol red. The cells were treated with hydrogen peroxide at various concentrations and the fluorescence (DPPP at Excitation: 351 nm, Emission: 380 nm or DCFDA at Excitation: 485 nm, Emission: 540 nm) was measured on a SpectraMax M3 plate reader from Molecular Devices (Sunnyvale, CA) after 4 hours.

### Peroxide Clearance

The rate of peroxide clearance was determined as described by Dringen and co-workers [31]. Briefly, the standard culture medium on a confluent astrocyte culture was replaced with incubation buffer (20 mM HEPES, 145 mM NaCl, 1.8 mM CaCl_2_, 5.4 mM KCl, 1 mM MgCl_2_, 0.8 mM Na_2_HPO_4_, and 5 mM glucose, pH 7.4). The cells were kept at 37°C and hydrogen peroxide was added to a final concentration of 200 µM. Aliquots of media (10 µl) were taken every minute for 6 minutes and added directly to 25 mM sulfuric acid in a 96-well plate. To these aliquots, 190 µl of reaction mixture (0.5 mM (NH_4_)_2_Fe(SO_4_)_2_, 200 µM xylenol orange and 200 mM sorbitol in 25 mM sulfuric acid) was added to each well. After incubating for 45 minutes, the hydrogen peroxide concentration was determined by comparing the absorbance (540 nm) of the samples versus a hydrogen peroxide concentration curve using a SpectraMax M3 plate reader.

### Protein Extraction for Quantitative Proteomics

After reaching confluence, the astrocytes were washed with phosphate buffered saline (PBS). Hypotonic lysis buffer (50 mM Tris-HCl at pH 7.4) was added to each flask and the cells were detached with a cell scraper and the resulting lysate was flash frozen in liquid nitrogen. The cell lysate was thawed and spun at 15,000 *g* for 3 minutes in a refrigerated centrifuge (4°C). The supernatant was removed and subjected to bicinchoninic acid (BCA) to determine protein concentration and each “light” labeled wild-type sample was combined with a “heavy” GFAP-Nrf2 sample at equal total protein amounts (n = 3 total).

### Protein Digestion for Quantitative Proteomics

Protein samples were diluted into 6 M urea/50 mM ammonium bicarbonate (pH 8). Cysteinyl disulfides were reduced via the addition of 2 mM Tris[2-carboxyethyl] phosphine (TCEP) for 30 minutes at 37°C. Reduced disulfides were then alkylated by the addition of 10 mM iodoacetamide (IAA) for 30 minutes in the dark. The urea was then diluted to <1 M with 50 mM ammonium bicarbonate, the sample concentrated via ultrafiltration (10 kDa cut-off), and the pH was adjusted to pH 8 and acetonitrile was added to 20%. Trypsin was added at a 1∶20 weight-to-weight ratio and incubated for 18 hours at 37°C. After digestion, the sample was dried by vacuum centrifugation.

### Offline High pH Reverse Phase Separation for 2D LC-MS/MS

100 µg of protein digest was reconstituted in 100 µl of 50 mM ammonium formate at pH 10. Peptides were separated by off-line high pH reverse phase using a Gemini C18 RP column (2×150 mm, 3 µM, 110 Å) from Phenomenex (Torrance, CA) with a 50 mM ammonium formate (pH 10) and acetonitrile mobile phase. Peptides were eluted with a linear gradient of acetonitrile from 5 to 35% over 60 minutes. Fractions were collected every 6 minutes for a total of 10 fractions. Each fraction was vacuum-centrifuged to dryness and then reconstituted in 30 µl of 0.1% formic acid and analyzed by LC-MS/MS.

### LC-MS/MS

Using a Waters NanoAcquity HPLC, tryptic peptides were separated with a 5–40% linear gradient of 0.1% formic acid in acetonitrile at a flow rate of 300 nl/min over 90 minutes. The eluted peptides were analyzed by a nanoelectrospray ionization (nESI) ion trap mass spectrometer (amaZon ETD) from Bruker Daltonics. The MS survey scan was performed in positive ion mode from *m*/*z* 400 to 2000, followed by data-dependent MS/MS using the Stable Isotope Pairs (SILE) acquisition method. The signal threshold for switching from the survey scan to MS/MS was set at 3000. Normalized collision energy was set at 35; capillary voltage, 3000 V; capillary temperature, 200°C. Dynamic exclusion was activated with the following parameters: repeat count was 1, repeat duration was 60 s, and the exclusion duration was 60 s.

### Database Search for Quantitative Proteomics

The result files (.*yep*) from each sample were searched using the Mascot Server (version 2.1.1) from Matrix Science. Using Mascot, the files were searched against the SwissProt database (UniProt) with the following parameters: taxonomy was limited to *mus musculus*, parent mass tolerance was 2.0 Da, fragment mass tolerance was 0.8 Da, and a maximum of two missed cleavages was allowed. Carbamidomethylation at cysteine residues was set as a fixed modification and oxidation of methionine was set as a variable modification. The false discovery rate (FDR) was determined to be less than 1% via the Mascot Search Engine.

### SILAC Quantitation, Functional Analysis, and Microarray Comparison

Expression differences between “heavy” (GFAP-Nrf2) and “light” (Wild-type) peptide pairs were quantified using Mascot Distiller from Matrix Science. Fold changes were exported to Microsoft Excel. Fold changes were converted to a log_2_ scale and then checked for a normal distribution via a histogram plot (**Figure S5)**. Peptide pairs were required to be identified in all three replicates and were only reported if they exhibited an average fold change of greater than +1.35 or less than −1.35. Only peptide pairs identified in all three SILAC replicates were included in the results. Functional analysis of the differentially expressed proteins was performed with the online tool DAVID Bioinformatics Resources 6.7 [71], [72]. Comparison against two previously published microarray data sets was performed [5], [7]. For the microarray data, each gene was required to have a fold change greater than 1.5 fold, a rank analysis score of at least 5, and a coefficient of variation of less than 1.

### siRNA Treatment

Confluent astrocytes were transfected with siRNA constructs with RNAiMAX Lipofectamine according to the manufacturer’s instructions (see **Table S1** for siRNA sequences). Briefly, each siRNA construct was diluted in Opti-MEM and then mixed with an equal volume of Lipofectamine diluted in Opti-MEM (1∶1 ratio Lipofectamine to siRNA) and allowed to incubate for 10 minutes. After incubation, the siRNA/Lipofectamine solution was added directly to cells without removing the original cell media. The final siRNA concentration was 25 nM. After 18 hours, the siRNA was removed and cells were infected with adenovirus or treated with tBHQ or vehicle.

### Quantitative PCR

After 24 hours of treatment vehicle control or tBHQ, astrocytes were rinsed once with PBS and total RNA was isolated from cells using TRIzol reagent according to manufacturer’s instructions. RNA quality was assessed with a Bioanalyzer 2100 from Agilent Technologies (Santa Clara, CA). The RNA Integrity Number (RIN) was required to be 8.5 or above. Reverse transcriptase was used to make cDNA using Oligo(dT)_15_ primers at 42°C for 1 hour. The quantitative PCR was performed with a LightCycler480 Real-Time PCR System from Roche. Briefly, Cycler480 SYBR Green I Master mix containing 1 µl of cDNA for each sample and 50 pmol of each primer were amplified (see **Table S2** for primer sequences).

### Western Blot Analysis

After 48 hours of treatment with adenovirus (adGFP or adNrf2) or chemical treatment (vehicle or tBHQ), astrocytes were rinsed once with PBS and then harvested in RIPA buffer. Protein concentration was assayed by bicinchoninic acid assay (BCA). Protein samples (10 µg) were resolved on a 12% SDS-PAGE gel and transferred to a Hybond-P membrane. The membrane was blocked with 5% non-fat milk in TBS with 0.1% Tween (TBS-T) for 1 hour before overnight incubation at 4°C with primary antibody. The following antibodies were used: polyclonal anti-GCLM (1∶10,000) a kind gift from Dr. Terrence Kavanaugh, rabbit monoclonal anti-Prdx6 (1∶2,000) from Epitomics (Burlingame, CA), rabbit monoclonal anti-Prdx1 (1∶10,000) from Epitomics, and mouse monoclonal anti-β-tubulin (1∶1,000) from the University of Iowa Developmental Studies Hybridoma Bank (Iowa City, IA). The membrane was incubated for 1 hour at room temperature with a horseradish peroxidase conjugated secondary antibody (1∶4000). The membrane was developed using an enhanced chemiluminescence (ECL) kit and then imaged and quantified using the G:Box imaging system from Syngene (Frederick, MD).

### Statistical Analysis

All data presented is reported as mean +/− standard deviation unless otherwise stated. All statistical computations were performed using GraphPad Prism 4.0 from GraphPad Software. Statistical significance was determined by using a Student’s t-test (p<0.05) or ANOVA (p<0.05) followed by a Newman-Keuls posthoc analysis to determine statistically significant paired comparisons (p<0.05).

## Supporting Information

Figure S1 **Adenoviral overexpression of Nrf2 and its effects on H_2_O_2_ toxicity.** Wild-type (WT) astrocytes were infected with adGFP or adNrf2 adenovirus and then treated with H_2_O_2_ as indicated. Cell viability was determined by **A)** MTS or **B)** LDH. Statistics were performed using 2-way ANOVA, * indicates p<0.01.
(TIF)

Figure S2 **The effect of GCLM siRNA knockdown on Nrf2 protection against 4-HNE.** adGFP or adNrf2 infected astrocytes were pretreated with **A)** non-targeting siRNA (siNT) or **B)** glutamate-cysteine ligase, modifier subunit siRNA (siGCLM). 4-HNE toxicity curves were performed as indicated. Cell viability was determined by MTS. Statistics were performed using 2-way ANOVA, * indicates p<0.01.
(TIF)

Figure S3 **The effect of sulfiredoxin-1 knockdown on cellular sensitivity to toxins and Nrf2 mediated protection.** Vehicle/tBHQ or adGFP/adNrf2 treated astrocytes were pretreated with either non-targeting (siNT) or sulfiredoxin-1 (siSrdxn) siRNA. Toxicity curves were performed as indicated: **A)** H_2_O_2_, **B)** tBOOH, or **C)** 4-HNE. Cell viability was determined by MTS. Statistics were performed using 2-way ANOVA, * indicates p<0.01.
(TIF)

Figure S4 **The effect of glutathione deficiency on extracellular H_2_O_2_ clearance.** Wild-type (WT) or glutamate-cysteine ligase, modifier subunit knockout (GCLM-KO) astrocytes were infected with adGFP or adNrf2 virus. The rate of H_2_O_2_ clearance from the extracellular medium was measured over time for wild-type (WT) or GCLM-knockout (GCLM-KO) cells. Statistics were performed using 2-way ANOVA, * indicates p<0.01.
(TIF)

Figure S5 **Log_2_ Histogram of SILAC Fold Change.** To check for a normal distribution of expression changes, raw fold changes for each protein were log_2_ converted, binned, and plotted.
(TIF)

Table S1 **siRNA Sequences**
(XLSX)

Table S2 **Primer Sequences for qPCR**
(XLSX)

Table S3 (XLSX)

Table S4 **Gene Ontologhy Analysis**
(XLSX)

Table S5 **Oxidative Stress Related Proteins**
(XLSX)
