# Supplementary figures and images for: Mechanisms of Nrf2 Protection in Astrocytes as Identified by Quantitative Proteomics and siRNA Screening
Source: PLoS One. 2013 Jul 29;8(7):e70163. doi: 10.1371/journal.pone.0070163 (PMC3726381; doi:10.1371/journal.pone.0070163)

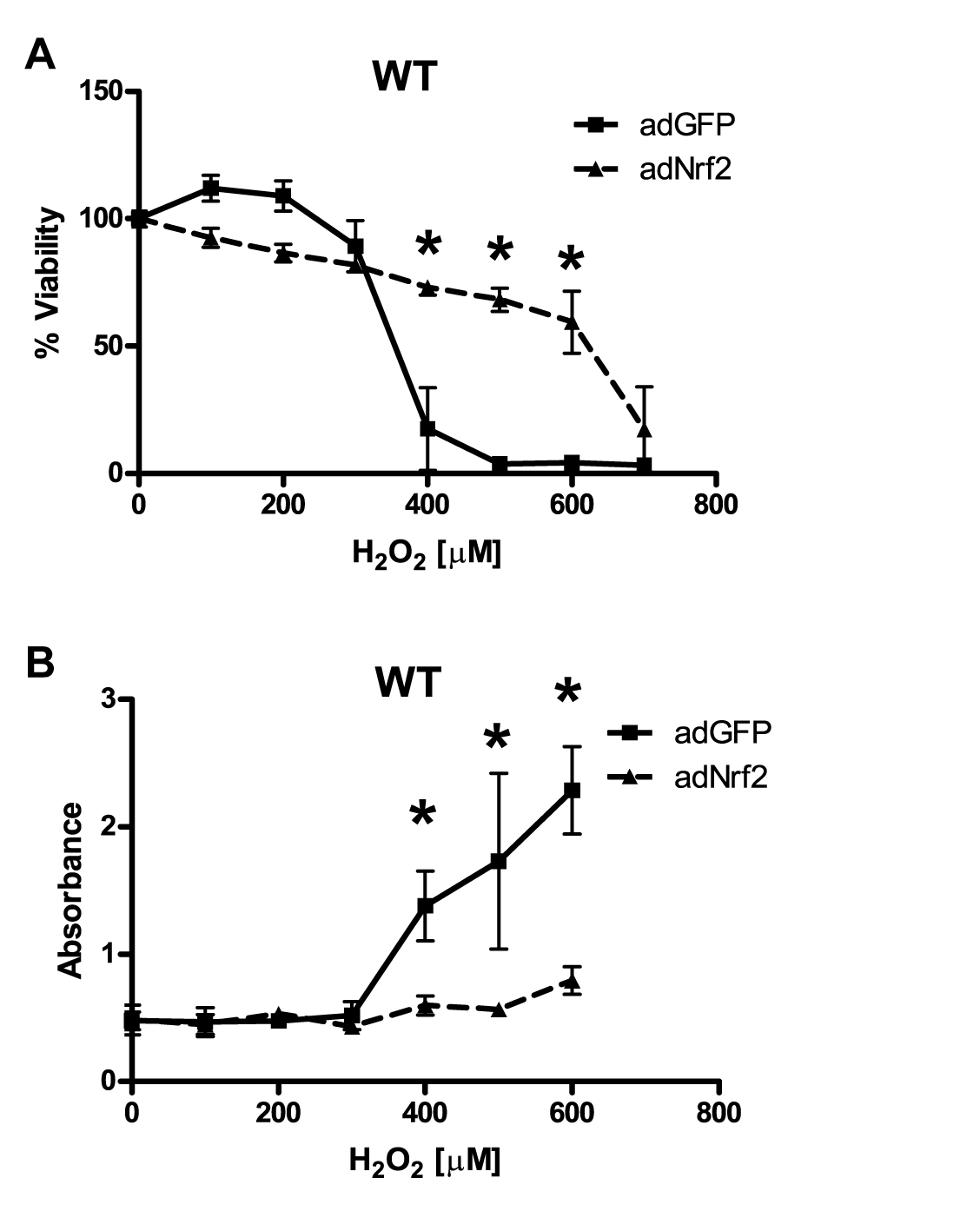

Supplement: Figure S1 — Adenoviral overexpression of Nrf2 and its effects on H2O2 toxicity. Wild-type (WT) astrocytes were infected with adGFP or adNrf2 adenovirus and then treated with H2O2 as indicated. Cell viability was determined by A) MTS or B) LDH. Statistics were performed using 2-way ANOVA, * indicates p<0.01. (TIF) [file pone.0070163.s001.tif]

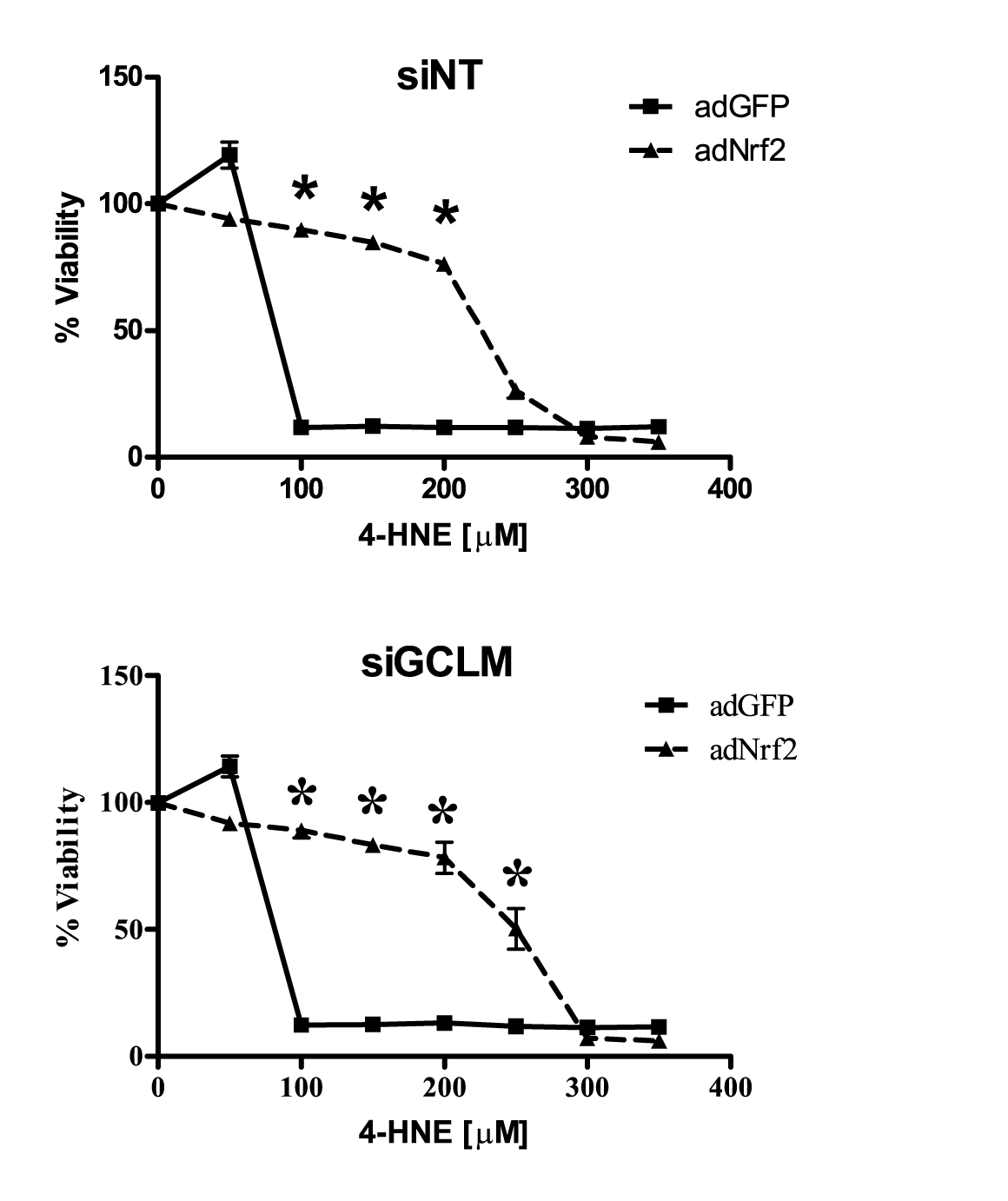

Supplement: Figure S2 — The effect of GCLM siRNA knockdown on Nrf2 protection against 4-HNE. adGFP or adNrf2 infected astrocytes were pretreated with A) non-targeting siRNA (siNT) or B) glutamate-cysteine ligase, modifier subunit siRNA (siGCLM). 4-HNE toxicity curves were performed as indicated. Cell viability was determined by MTS. Statistics were performed using 2-way ANOVA, * indicates p<0.01. (TIF) [file pone.0070163.s002.tif]

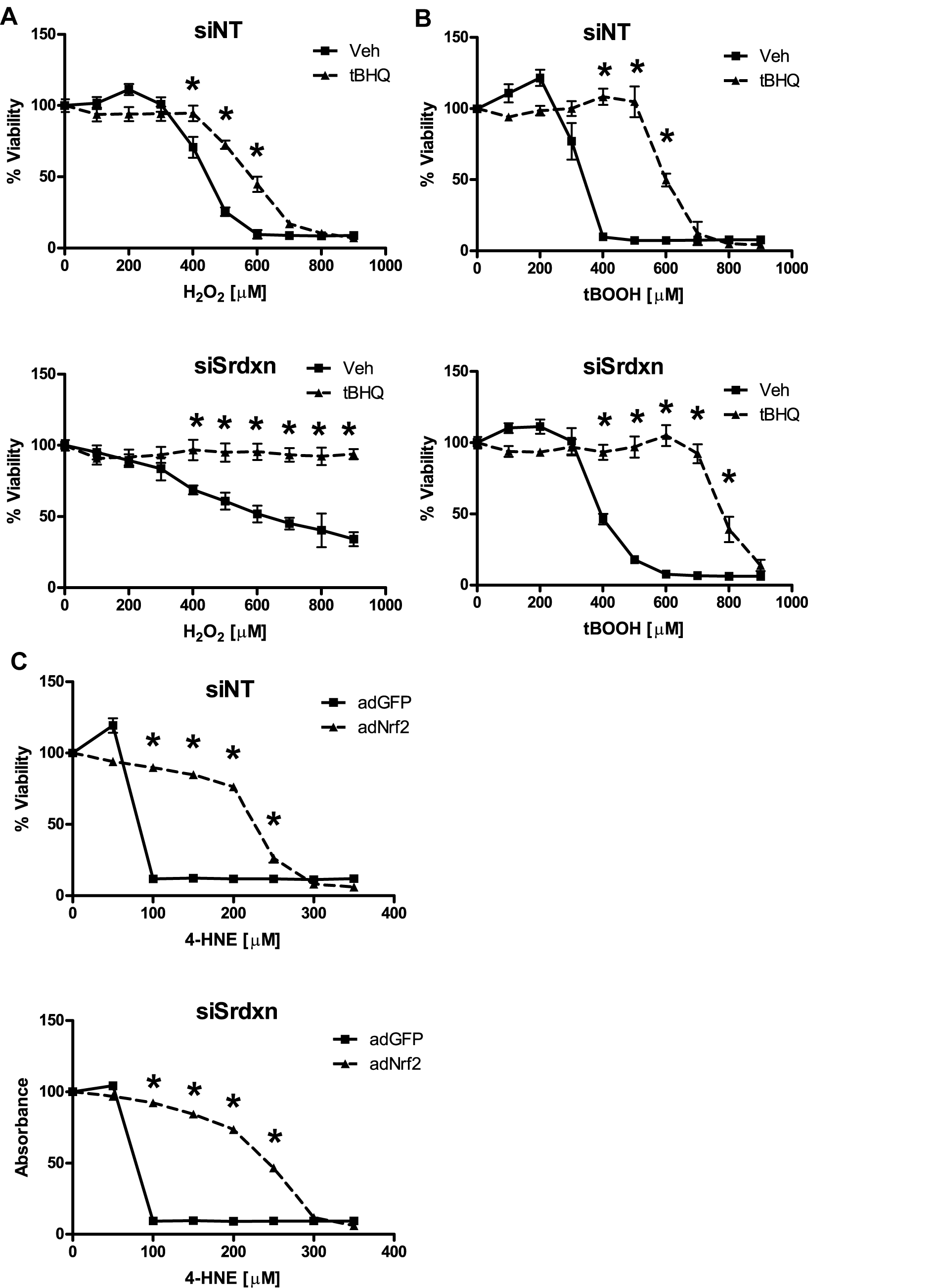

Supplement: Figure S3 — The effect of sulfiredoxin-1 knockdown on cellular sensitivity to toxins and Nrf2 mediated protection. Vehicle/tBHQ or adGFP/adNrf2 treated astrocytes were pretreated with either non-targeting (siNT) or sulfiredoxin-1 (siSrdxn) siRNA. Toxicity curves were performed as indicated: A) H2O2, B) tBOOH, or C) 4-HNE. Cell viability was determined by MTS. Statistics were performed using 2-way ANOVA, * indicates p<0.01. (TIF) [file pone.0070163.s003.tif]

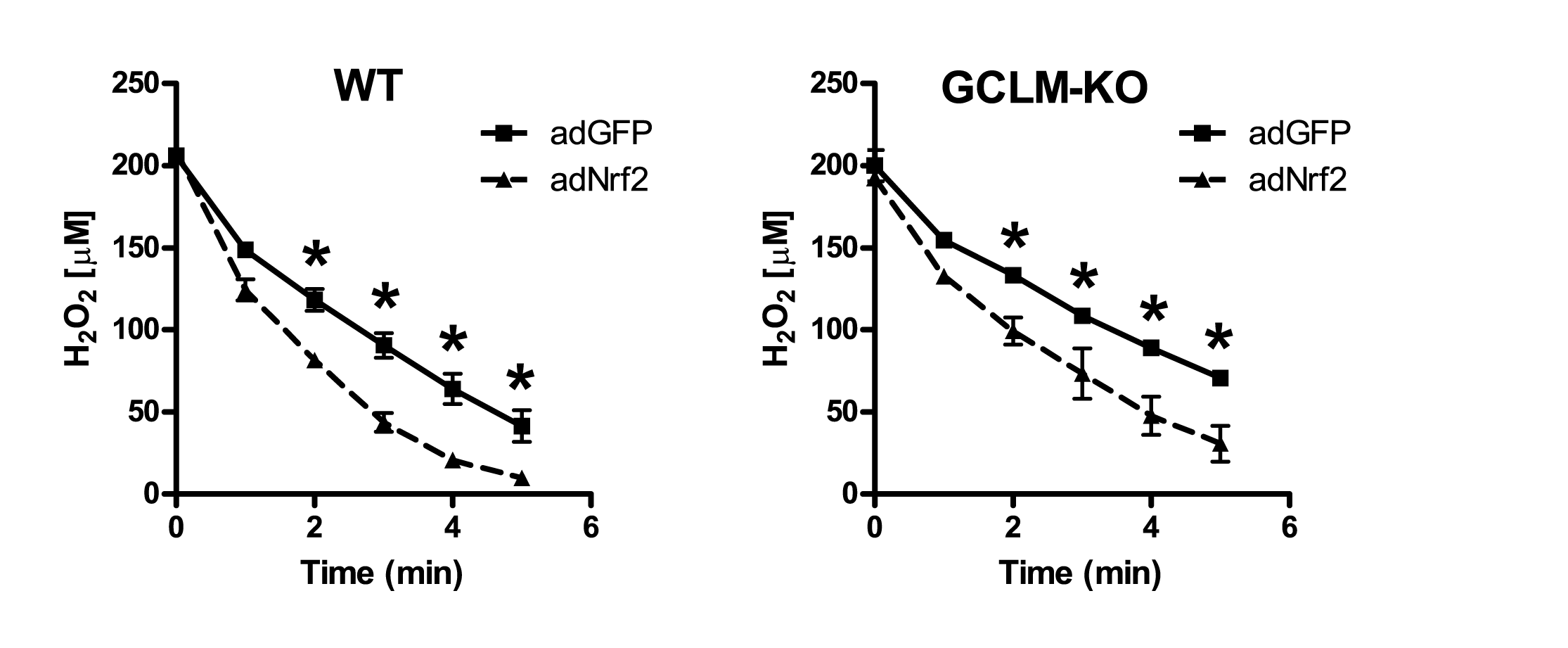

Supplement: Figure S4 — The effect of glutathione deficiency on extracellular H2O2 clearance. Wild-type (WT) or glutamate-cysteine ligase, modifier subunit knockout (GCLM-KO) astrocytes were infected with adGFP or adNrf2 virus. The rate of H2O2 clearance from the extracellular medium was measured over time for wild-type (WT) or GCLM-knockout (GCLM-KO) cells. Statistics were performed using 2-way ANOVA, * indicates p<0.01. (TIF) [file pone.0070163.s004.tif]

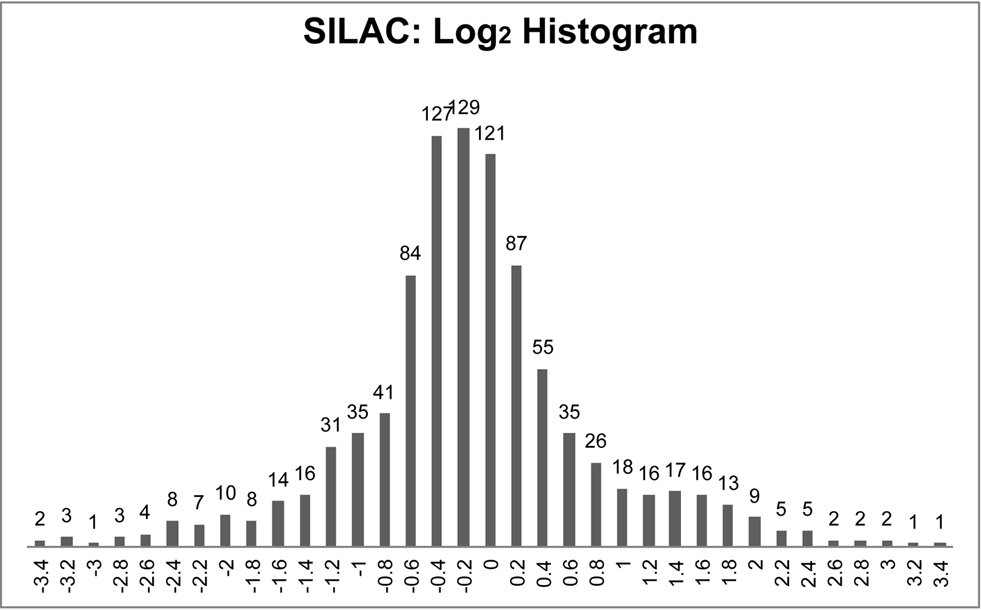

Supplement: Figure S5 — Log2 Histogram of SILAC Fold Change. To check for a normal distribution of expression changes, raw fold changes for each protein were log2 converted, binned, and plotted. (TIF) [file pone.0070163.s005.tif]
